# Condensin II Regulates Interphase Chromatin Organization Through the Mrg-Binding Motif of Cap-H2

**DOI:** 10.1534/g3.115.016634

**Published:** 2015-03-09

**Authors:** Heather A. Wallace, Joseph E. Klebba, Thomas Kusch, Gregory C. Rogers, Giovanni Bosco

**Affiliations:** * Department of Genetics, Geisel School of Medicine at Dartmouth, Hanover, New Hampshire 03755; † Department of Cellular and Molecular Medicine, University of Arizona Cancer Center, Tucson, Arizona 85721; ‡ Somerset, New Jersey 08873

**Keywords:** chromatin organization, Mrg15, chromosome structure, condensin, homolog pairing

## Abstract

The spatial organization of the genome within the eukaryotic nucleus is a dynamic process that plays a central role in cellular processes such as gene expression, DNA replication, and chromosome segregation. Condensins are conserved multi-subunit protein complexes that contribute to chromosome organization by regulating chromosome compaction and homolog pairing. Previous work in our laboratory has shown that the Cap-H2 subunit of condensin II physically and genetically interacts with the *Drosophila* homolog of human MORF4-related gene on chromosome 15 (MRG15). Like Cap-H2, Mrg15 is required for interphase chromosome compaction and homolog pairing. However, the mechanism by which Mrg15 and Cap-H2 cooperate to maintain interphase chromatin organization remains unclear. Here, we show that Cap-H2 localizes to interband regions on polytene chromosomes and co-localizes with Mrg15 at regions of active transcription across the genome. We show that co-localization of Cap-H2 on polytene chromosomes is partially dependent on Mrg15. We have identified a binding motif within Cap-H2 that is essential for its interaction with Mrg15, and have found that mutation of this motif results in loss of localization of Cap-H2 on polytene chromosomes and results in partial suppression of Cap-H2-mediated compaction and homolog unpairing. Our data are consistent with a model in which Mrg15 acts as a loading factor to facilitate Cap-H2 binding to chromatin and mediate changes in chromatin organization.

Within the eukaryotic nucleus, DNA is packaged into chromatin that is spatially organized in a nonrandom manner (Cremer and Cremer 2010; Kosak and Groudine 2004; Misteli 2007). Dynamic nuclear organization of chromatin within the interphase nucleus facilitates precise coordination of essential cellular processes, including transcription, DNA replication, DNA repair, and chromosome segregation (Belmont 2006; Jackson 2010; Laster and Kosak 2010; Macalpine and Almouzni 2013; Thadani *et al.* 2012). Interphase chromosomes are arranged into discrete subnuclear regions termed chromatin territories, functionally partitioning the genome into active or repressive compartments (Cremer and Cremer 2001, 2010; Fraser and Bickmore 2007; Lieberman-Aiden *et al.* 2009) and underscoring the relationship between nuclear organization and function. Understanding how chromatin is organized within the three-dimensional (3D) space of the nucleus is essential for understanding the regulation of the various nuclear processes influenced by chromatin architecture.

Condensins are conserved multi-subunit protein complexes that are well-known for their roles in mitotic chromosome condensation and segregation (Hirano *et al.* 1997). Eukaryotes have two highly conserved condensin complexes, condensin I and condensin II. These complexes consist of two heterodimeric core Structural Maintenance of Chromosome (SMC) proteins, SMC2 and SMC4, which contain an ATPase “head” domain and a “hinge” domain required for dimerization (Anderson *et al.* 2002; Hirano and Hirano 2006). Condensins I and II each posses unique non-SMC Chromosome-Associated Protein (CAP) subunits. Condensin I contains Cap-H, Cap-D2 and Cap-G, whereas condensin II contains Cap-H2, Cap-D3, and Cap-G2, although no Cap-G2 homolog has been identified in *Drosophila* (Neuwald and Hirano 2000; Ono *et al.* 2003). The two condensin complexes possess both localization and functions that are distinct from one another, with condensin I promoting lateral compaction of chromosomes, whereas condensin II promotes axial compaction (Bauer *et al.* 2012; Buster *et al.* 2013; Green *et al.* 2012; Hirota *et al.* 2004; Shintomi and Hirano 2011). Whereas condensin I localizes to the cytoplasm and is only found on chromosomes after nuclear envelope breakdown during prometaphase, condensin II localizes to the nucleus throughout the cell cycle (Hirota *et al.* 2004; Ono *et al.* 2004).

The distinct spatial and temporal localization pattern of condensin complexes suggests that condensin II plays an important role in organization of the interphase nucleus, and a number of recent studies provide support for its involvement in numerous processes during interphase (Wallace and Bosco 2013). For example, *Drosophila* condensin II functions as an anti-pairing factor that antagonizes pairing of homologous chromosomes and transvection (Bateman *et al.* 2012; Bauer *et al.* 2012; Joyce *et al.* 2012). The anti-pairing activity of condensin II is proposed to be a direct consequence of its function in promoting axial compaction of chromosomes, which drives the spatial reorganization of interphase chromosomes into chromosome territories that occupy distinct regions within the nucleus (Bauer *et al.* 2012; Hartl *et al.* 2008; Joyce *et al.* 2012). Control of nuclear architecture has important implications for gene expression, and several lines of evidence exist linking condensin complex activity with gene regulation (Cobbe *et al.* 2006; Csankovszki *et al.* 2009; Dej *et al.* 2004; Jans *et al.* 2009; Longworth *et al.* 2008; Zhang *et al.* 2010), including the observation that the Cap-D3 subunit of condensin II is important for regulation of expression of immunity genes and represses transposon activation in *Drosophila* (Longworth *et al.* 2012; Schuster *et al.* 2013). Additionally, condensin-mediated compaction of chromatin promotes mammalian erythroid cell differentiation as well as T-cell quiescence (Rawlings *et al.* 2011; Xu *et al.* 2006). More recently, condensin II has been shown to bind active promoters and enhancers in both *C. elegans* and in mouse embryonic stem cells (Dowen *et al.* 2013; Kranz *et al.* 2013). However, the mechanisms by which condensin II regulates both global genome organization and local gene transcription remain unclear.

We have previously shown that *Drosophila* Cap-H2 interacts with the *Drosophila* homolog of the MORF4-related gene on chromosome 15 (Mrg15) via the MRG domain of Mrg15 (Smith *et al.* 2013). Mrg15 is a highly conserved chromodomain protein that plays roles in a number of fundamental cellular processes, including cell proliferation, DNA repair, and regulation of gene expression (Garcia *et al.* 2007; Hayakawa *et al.* 2010; Leung *et al.* 2001; Tominaga *et al.* 2005; Zhang *et al.* 2010). Mrg15 binds to methylated H3K36 and H3K4, histone marks correlated with transcriptional activation (Joshi and Struhl 2005; Moore *et al.* 2010; Zhang *et al.* 2006a). Mrg15 is a component of several complexes associated with transcriptional regulation, including the Tip60 HAT complex and the mammalian Sin3/HDAC chromatin remodeling complex (Kusch *et al.* 2004; Pardo *et al.* 2002; Yochum and Ayer 2002). Similar to Cap-H2, Mrg15 is required for condensin II–mediated chromosome unpairing, suppression of transvection, and axial compaction of chromosomes (Smith *et al.* 2013). Furthermore, binding of Cap-H2 to chromatin is partially dependent on Mrg15, suggesting that Mrg15 may recruit Cap-H2 to chromatin to facilitate condensin II activity on interphase chromosomes (Smith *et al.* 2013).

To better understand the mechanisms by which Cap-H2 and Mrg15 function together to modulate chromatin organization, we further assessed the interaction between these two proteins and analyzed their localization on chromatin. Here, we show that Cap-H2 localizes to interband regions on polytene chromosomes in a manner partially dependent on Mrg15. We have identified an Mrg15 binding consensus sequence motif within Cap-H2 that is essential for its interaction with Mrg15, and we have found that mutation of this motif partially suppresses Cap-H2-mediated compaction and homolog unpairing. Consistent with ChIP-seq data describing condensin binding profiles in other organisms, we observed that Cap-H2 co-localizes with Mrg15 at regions of active transcription, suggesting that Cap-H2 may play a role in transcription. We propose a model in which Mrg15 acts as a loading factor to facilitate Cap-H2 binding to chromatin and mediate changes in chromatin organization.

## Materials and Methods

### *Drosophila* stocks

All fly stocks were cultured on standard cornmeal/molasses cornmeal/agar media with yeast at 25°. *Oregon-R*, *yw (y^1^w^67c23^*) and *Mrg15 RNAi* (y^1^ sc* v^1^; *P{TRiP.GL00128}attP2/TM3*, *Sb^1^*) stocks were from Bloomington Stock Center. w[*]; P(w[+mC] = lacO.256x)60F, *hs83 > GFP-LacI*; *Hsp70 > Gal4*, *Cap‐H2^EY09979^* (Cap-H2 pairing reporter line) and Cap-H2^Z3-0019^ and ru h st Cap-H2^Z3-0019^ sr e ca/TM6B, Hu Tb e ca (*Cap-H2* mutant) were described previously (Hartl *et al.* 2008).The *43B-Gal4* driver was obtained from Patrick O’Farrell, personal communication.

### cDNA constructs and transgenesis

pMT-Cap-H2-eGFP and pMT-Cap-H2-ΔC23-eGFP constructs were made as described previously (Buster *et al.* 2013). Site-directed mutagenesis was performed using QuikChange II (Agilent Technologies) to generate pMT-Cap-H2-MBM-eGFP and pMT-Cap-H2-MBM-ΔC23-eGFP plasmids. Cap-H2-eGFP and Cap-H2-MBM-eGFP sequences were subcloned into the pUAST-attB vector to generate the UAS-Cap-H2-eGFP and UAS-Cap-H2-MBM-eGFP constructs, which were injected into *y^1^w^67c23^*; *P{CaryP}attP2* embryos by Best Gene Inc.

### Cell culture, transfection, and RNAi

S2 and Kc167 cells were cultured at 25° in Sf900II media (Life Technologies) supplemented with 1× Antibiotic-Antimycotic (Gibco). Transient transfections were performed using the Nucleofector II (Lonza) according to manufacturer’s instructions. At 24 hr after transfection, expression of all constructs was induced by addition of 1 mM CuSO_4_ for 24 hr. RNAi treatments were performed in six-well tissue culture dishes (Olympus Plastics) or 10-cm^2^ tissue culture dishes (Corning) by addition of 10 µg/mL double-stranded RNA (dsRNA) to cells plated at 70–90% confluency. Wells were replenished with 10 µg/mL dsRNA in fresh media every other day for 5 d and cells were harvested on day 6. dsRNA was made using gene-specific primer sequences as described previously (Smith *et al.* 2013). The PCR product used to generate control (SK) dsRNA was amplified using the pEGFP-N1 vector (Takara Bio Inc.) as template. PCR products were then used as templates to generate dsRNA with the T7 RiboMAX Express Large Scale RNA Production System kit (Promega). dsRNA concentration was then calculated using gel electrophoresis and densitometry analysis (ImageJ).

### Co-immunoprecipitations and immunoblots

For GFP immunoprecipitations, GFP-binding protein was bound to Protein A-coupled Sepharose resin as described previously (Buster *et al.* 2013). GBP-coated beads were washed three times with 1.5 ml of cell lysis buffer (CLB; 100 mM Tris, pH 7.2, 125 mM NaCl, 1 mM DTT, 0.1% Triton X-100, and 0.1 mM PMSF). Expression of Cap-H2-EGFP or Cap-H2-MBM-EGFP in transfected cells was induced with 1–2 mM CuSO_4_. After 24 hr, transfected cells were lysed in CLB, clarified by centrifugation, and then lysates were diluted to 2–5 mg/ml in CLB. Antibody-coated beads were mixed with lysate for 40 min at 4°, washed three times with CLB, and then boiled in Laemmli sample buffer. Cap-H2-EGFP and Cap-H2-EGFP was detected on immunoblots with mouse monoclonal anti-GFP (JL-8; Clontech). Primary antibodies for immunoblots of dsRNA-treated cells were guinea pig anti-Mrg15 (1:500, gift from Tom Kusch, personal communication) and rabbit anti-Kinesin Heavy Chain (1:1000, Cytoskeleton). Guinea pig anti-Mrg15 serum was generated as described previously (Kusch *et al.* 2004).

### Immunostaining and quantification

Kc cells were plated on Concanavalin A (Con-A)–coated cover slips to allow cells to adhere and fixed with 4% formaldehyde in PBS at room temperature. Fixed cells were permeabilized with phosphate-buffered saline (PBS) with 0.1% Triton X-100 (PBT) and blocked in buffer containing PBT and 5% normal goat serum (Sigma). The following primary antibodies were used: rabbit anti-Cid (1:500) (Buster *et al.* 2013), mouse anti-GFP (1:250, JL-8; Living Colors), rabbit anti-Cap-H2 serum (1:50) (Hartl *et al.* 2008), rabbit anti-Cap-D3 (1:50, gift from Michelle Longworth, personal communication) (Longworth *et al.* 2008) and guinea pig anti-Mrg15 serum (1:250, gift from Tom Kusch). Secondary antibodies were conjugated with Alexa488, Cy2, FITC, or Cy3 (1:500, Jackson ImmunoResearch Laboratories). 4′,6-diamidino-2-phenylindole (DAPI) was used at a final concentration of 0.1 μg/μl. Stained cells were mounted in Vectashield Mounting medium (Vector Laboratories) and imaged using a Nikon A1RSi confocal microscope with Plan Apo 60× and 100× oil immersion objectives and the Nikon Elements 4.0 software package. Images were processed using Nikon Elements. Cid spot counts were performed on maximum z-projections from z-stack images using the counting software in Nikon Elements.

Salivary gland squashes were performed as previously described (Wallace *et al.* 2010). Briefly, salivary glands were dissected in 0.7% NaCl, placed onto cover slips coated with Repel-Silane (GE Healthcare) and fixed in a solution containing 3.7% formaldehyde in 45% acetic acid. Fixed salivary glands were then inverted onto a microscope slide coated with poly-L-lysine (Sigma) and squashed. Glands from control and experimental genotypes were squashed and stained side-by-side to compare fluorescence intensities. Slides were frozen in liquid nitrogen. Primary antibodies were diluted in PBS containing 0.1% Nonidet P-40 (NP-40) and 1% nonfat milk and added to slides that were incubated in a humid chamber at 4° overnight. Slides were washed in PBS containing 0.1% NP-40 for 5 min and incubated with secondary antibodies for 2 hr in humid chamber at RT. Slides were then washed in PBS, stained with DAPI (0.1 μg/μl), and mounted in Vectashield Mounting medium (Vector Laboratories). The following primary antibodies were used: rabbit anti-Cap-H2 (1:50) (Hartl *et al.* 2008), and guinea pig anti-Mrg15 serum (1:100, gift from Tom Kusch), mouse anti-GFP (1:100, JL-8; Living Colors), rabbit anti-H3K36me3 (1:250, ab9050; Abcam), and mouse anti-Pol II (1:100, ab5131; Abcam).

To compare fluorescence intensities of proteins on polytene chromosomes from control and experimental genotypes, images were captured using the same exposure time and processed under identical conditions. To quantify relative fluorescence intensities, at least four representative maximum projections consisting of the same number of confocal sections from each of three independent experiments were analyzed. Fluorescence was quantified by measuring pixels within a region of interest (ROI) corresponding to the polytene chromosomes of a single nucleus in ImageJ (NIH). Background fluorescence was measured outside the ROI and was subtracted from the measured fluorescence within the ROI. Results were normalized to background subtracted DAPI fluorescence values from the same ROI. *P* values were calculated using a two-tailed, unpaired Student's *t*-test.

For analysis of co-localization, immunostaining signals from each fluorescent channel were compared as described previously (Nowak *et al.* 2012). Briefly, 100 Cap-H2 immunostaining bands were counted from three independent nuclei and were considered as co-localized when they overlapped with a signal in the channel corresponding to Mrg15 or Pol II staining. Data were plotted using Microsoft Excel.

### Fluorescent *in situ* hybridization

Fluorescent *in situ* hybridization (FISH) in Kc cells was performed as previously described (Buster *et al.* 2013; Joyce *et al.* 2012; Smith *et al.* 2013). Cells were plated onto Con-A-coated cover slips in a well of a six-well tissue culture plate to allow cells to adhere. Cover slips were then washed with PBS and fixed in 4% formaldehyde in PBS for 10 min at room temperature (RT). Fixed cells were permeabilized with PBT and washed in CSK buffer [10 mM HEPES, 100 mM NaCl, 3 mM MgCl_2_, 300 mM sucrose, and phenylmethanesufonyl fluoride (PMSF)] for 10 min, followed by treatment with RNase A (100 ug/mL, ThermoScientific) for 1 hr at RT. Cells were then washed with 0.1 N HCl for 5 min and dehydrated in a graded ethanol series: 70%, 90%, and 100% (5 min each). Cells were washed with 2× saline-sodium citrate (SSC) with 0.1% Triton X-100 (SSCT) and pre-hybridization buffer (50% formamide in 2× SSCT) was added for 2 hr at 37°; 1 µL of each FISH probe was added to 25 µL hybridization solution (1:1.5:5 mixture of dextran sulfate/20× SSC/formamide). The probe mixture was denatured at 95° for 2 min, snap-cooled on ice, added onto a microscope slide, and covered with a cover slip. Slides were then sealed with rubber cement, denatured at 93° on a heat block for 3 min, and placed in a humid chamber overnight at 37°. After hybridization, cover slips were detached from slides by immersing in 2× SSCT plus 50% formamide with shaking for 10 min and then placed into six-well tissue culture plates and washed in 2× SSCT plus 50% formamide three times for 30 min at 42°. Two 10-min washes were then performed at 42° with 40% and 20% formamide in 2× SSCT, respectively, followed by three 2× SSCT washes for 5 min at RT. Cells were stained with DAPI (0.1 μg/μl) in PBS for 10 min at RT, washed two times for 10 min at RT in PBS, and then mounted in Vectashield Mounting medium. Cells were imaged using a Nikon A1RSi confocal microscope with Plan Apo 60× and 100× oil immersion objectives and the Nikon Elements 4.0 software package with z-slices of 0.3 μm. FISH spot counts were performed on maximum projections from z-stack images using the counting software in Nikon Elements. The 3D FISH distance measurements were performed manually in Nikon Elements using the 3D distance-measuring tool by scanning through each z-slice. The centroid of each FISH signal was marked and the shortest 3D pairwise distance was measured as described previously (Lau *et al.* 2014). Statistical analyses were performed in Microsoft Excel using Student's *t*-tests.

### FISH probe preparation

Euchromatic FISH probes were made as previously described (Buster 2013; Smith 2013) from BAC clones (CHORI BACPAC Resources) as follows: X1, BACR30C13 and BACR18F10; X2, BACR20K01 and BACR35A18; 2L (1), BACR30M19 and BACR29P12; and 2L (2), BACR14I17 and BACR15P08. BAC clones were mapped and picked using the UCSC genome browser (genome.ucsd.edu). Clones were cultured, DNA was purified using the Plasmid Midi Kit (Qiagen), and purified DNA was amplified using the Whole Genome Amplification kit (Sigma). Amplified DNA (µg) was digested using a restriction enzyme cocktail consisting of AluI, Rsa, *Mse*I, *Msp*I, *Hae*III, and BfuCl (New England BioLabs) overnight at 37° and then ethanol-precipitated. DNA was denatured at 100° for 1 min and 3′-end-labeled with aminoallyl dUTP and terminal deoxynucleotidyl transferase (Roche) for 2 hr at 37°. Five mM EDTA was added to terminate the reaction and, after ethanol precipitation, DNA was resuspended in 10 uL ddH_2_O and conjugated to fluorophores using ARES Alexa Fluor DNA labeling kits (Invitrogen) according to the manufacturer’s instructions. Probes were then cleaned using Qiagen PCR clean-up kit (Qiagen), ethanol-precipitated, and resuspended in 10 µL EB buffer (Qiagen).

### Salivary gland pairing

The salivary gland polytene pairing assays were performed with a transgenic line containing a 256-repeat array of the Lac-O sequence at chromosomal position 60F and carrying a heat shock–inducible transgene Hs > GFP-LacI, which encodes a fluorescent fusion protein that binds to the LacO arrays and marks the chromosomal insertion site of the LacO array (Vazquez *et al.* 2001). This stock also contains Hsp70 > Gal4 and UAS > Cap-H2 transgenes, as described previously (Hartl *et al.* 2008). Homozygotes of this stock were crossed with homozygotes of the UAS-Cap-H2-GFP or UAS-Cap-H2-MBM-GFP stocks. Expression of GFP-LacI, Cap-H2, and Cap-H2-GFP or Cap-H2-MBM-GFP was induced with heat shock at 32° for 16 hr, followed by a 2.5-hr recovery at 25°. Salivary glands from third instar larvae were dissected in PBS with 0.1% Triton X-100 (PBT), and glands were fixed for 10 min in PBS plus 4% formaldehyde at RT. Glands were rinsed three times with PBT, stained for 10 min with 0.1 µg/μl DAPI in PBS, and then washed twice with PBS for 5 min. Glands were mounted in Vectashield Mounting medium (Vector Laboratories) and imaged using a Nikon A1RSi confocal microscope with Plan Apo 60× objective and the Nikon Elements 4.0 software package with z-slices of 0.3 μm. The number of GFP spots per nucleus was counted manually from maximum z-projections from z-stack images using the counting software in Nikon Elements. Two different biological replicates were imaged for the GFP spot quantification, and five nuclei from each of at least four glands per replicate were analyzed. *P* values were calculated using a two-tailed, unpaired Student's *t*-test.

### Chromatin immunoprecipitation and qPCR

Cells were cultured in T-75 tissue culture flasks (Thermo Scientific) and 50 million cells were used per immunoprecipitation. Cells were fixed with 1% formaldehyde for 10 min at room temperature with mixing. Glycine was added to a final concentration of 0.125 M. Cells were harvested by scraping, pelleted, and washed twice with ice-cold PBS. Cells were then resuspended in 10 mL cold lysis buffer [5 mM PIPES (pH 8.0), 85 mM KCl, 0.5% NP-40] with Complete EDTA-free protease inhibitor cocktail (Roche) and incubated for 10 min on ice. Cells were pelleted and nuclei were resuspended in 1 mL nuclei lysis buffer [50 mM Tris-Cl (pH 8.0), 10 mM EDTA, 1% SDS] with protease inhibitors. Samples were sonicated at 35% amplitude 10 times for 10 sec each, followed by 30 sec on ice with a Branson SLPe Digital Sonifier (Branson Ultrasonics Corporation). Samples were centrifuged at 14,000 rpm at 4° for 15 min; 100 ug of chromatin was used for each immunoprecipitation and diluted in 300 μl dilution buffer [16.7 mM Tris-Cl (pH 8.0), 167 mM NaCl, 1.2 mM EDTA, 0.01% SDS, 1.1% Triton X-100] with protease inhibitors. Thirty μl (10%) was set aside as input. Samples were incubated at 4° overnight with rabbit anti-Cap-H2 (1:50) (Hartl *et al.* 2008). Chromatin/antibody complexes were captured by incubation with Protein A/G Magnetic Beads (Pierce) for 2 hr at 4° with rotation. The beads were washed four times with high-salt wash buffer [50 mM HEPES (pH 7.9), 500 mM NaCl, 1 mM EDTA, 0.1% SDS, 1% Triton X-100, 0.1% deoxycholate] and two times with Tris-EDTA [10 mM Tris-HCl, (pH 8.0), 1 mM EDTA (pH 8.0)] at room temperature. Input samples and beads were resuspended in 300 μl of elution buffer [50 mM Tris-Cl (pH 8.0), 10 mM EDTA, 1% SDS] supplemented with 1 μl 20 μg/μl Proteinase K (Boehringer Mannheim) and crosslinks were reversed by overnight incubation at 65°. DNA was purified by phenol/chloroform extraction using Phase Lock Gel tubes (5 PRIME). Real-time PCR was performed using iTaq Universal SYBR Green Supermix (BioRad) on the Step-One Plus Real-Time PCR system (Applied Biosystems). RpL49 was used as internal control. Supporting Information, Table S1 shows primers used for qPCR. Enrichment of immunoprecipitated DNA fragments from four independent biological replicates was calculated using the 2^−∆∆Ct^ method based on the threshold cycle (Ct) value for each PCR reaction (Livak and Schmittgen 2001). Results are presented as percentage of total input of IP relative to background (IgG control). *P* values were determined using a two-tailed, unpaired Student's *t*-test.

## Results

### Cap-H2 and Mrg15 bind to transcriptionally active regions of the genome

**Figure 1:**
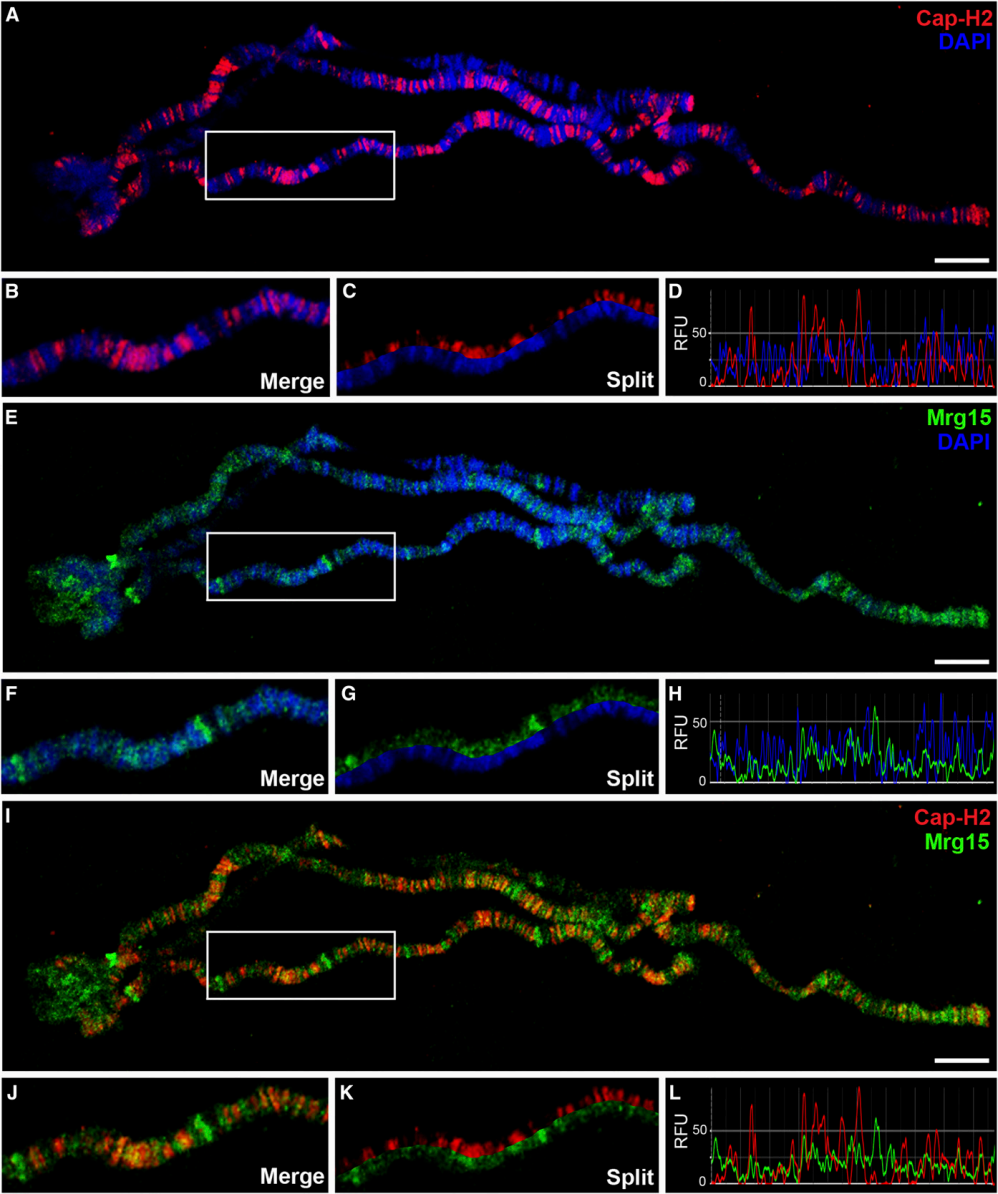
Cap-H2 co-localizes with Mrg15 at interbands of salivary gland polytene chromosomes. (A, E, I) Salivary gland polytene chromosomes from wild-type larvae stained for Cap-H2 (red), Mrg15 (green), and DNA (blue) as indicated. Scale bar, 10 μm. Enlarged merged (B) and split (C) image of boxed area in (A) showing Cap-H2 staining at interband regions. (D) Fluorescence intensity plots of image in (B) showing Cap-H2 and DAPI peaks do not overlap. Enlarged merged (F) and split (G) image of boxed area in (E) showing Mrg15 staining at interband regions. (H) Intensity plots of image in (F) showing Mrg15 and DAPI peaks do not overlap. Enlarged merged (J) and split (K) image of boxed area in I showing co-localization of Cap-H2 and Mrg15. (L) Fluorescence intensity plots of image in (J) showing frequent overlap of Mrg15 and Cap-H2 peaks. RFU, relative fluorescence units.

We have previously shown in chromatin fractionation experiments that global levels of Cap-H2-GFP on chromatin are partially dependent on Mrg15, suggesting that Mrg15 recruits the Cap-H2 protein to chromatin (Smith *et al.* 2013). To gain additional insight into effects of Mrg15 on the location of endogenous Cap-H2 on interphase chromatin, we first analyzed the localization of Cap-H2 and Mrg15 on larval salivary gland polytene chromosomes. Polytene chromosomes allow for direct visualization of chromatin-associated proteins at relatively high resolution, therefore providing a useful system for cytological analysis of chromatin organization of interphase nuclei. We first confirmed specificity of the guinea pig anti-Mrg15 antibody by Western blotting of lysates from control and Mrg15 RNAi-treated Kc cells (Figure S1). We performed immunofluorescence using antibodies against endogenous Cap-H2 and Mrg15 proteins on polytene chromosomes from salivary glands of third-instar larvae and detected the presence of Cap-H2 at hundreds of euchromatic sites (Figure 1A). Cap-H2 predominantly localizes to interband regions, which are weakly stained with DAPI (Figure 1, B–D). Mrg15 shows a similar pattern of localization to interband regions (Figure 1, E–H) and the Cap-H2 immunofluorescence signal co-localizes extensively with that of Mrg15 (90.3% overlap) (Figure 1, I–L and Figure S2).

**Figure 2:**
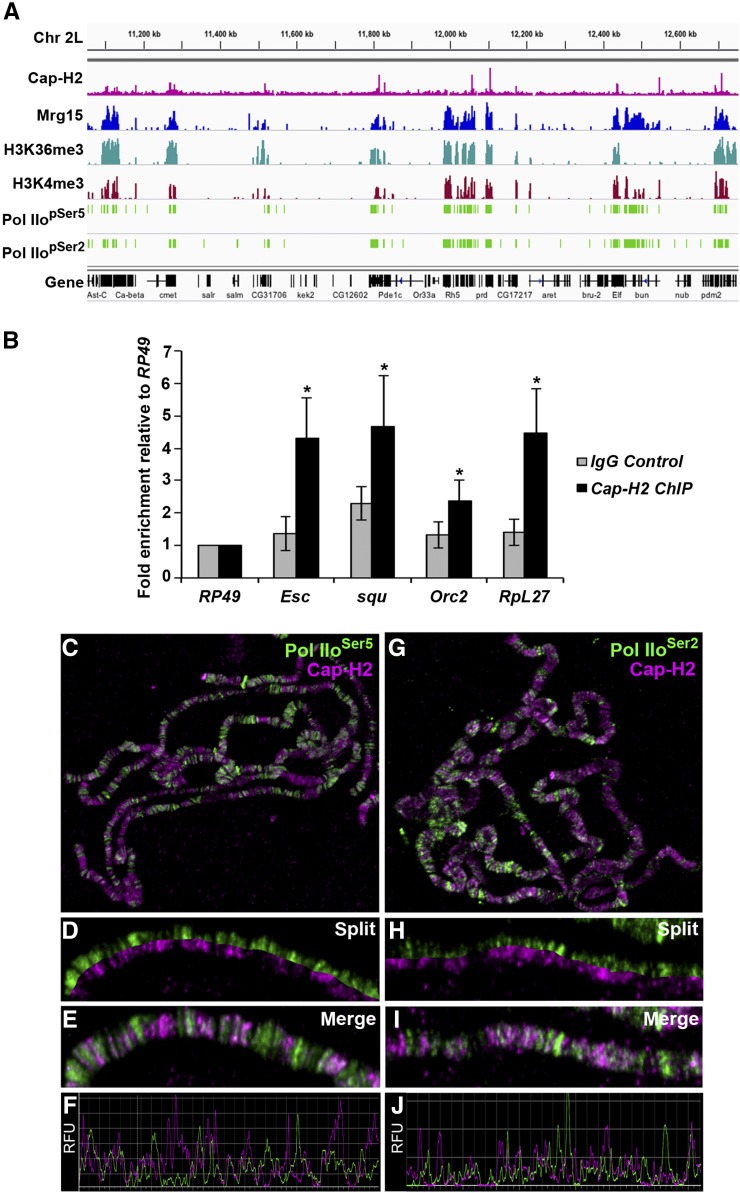
Cap-H2 binding overlaps with Mrg15 at transcriptionally active regions. (A) Representative Integrated Genome Viewer (IGV) screenshot of ChIP-seq binding profiles for Cap-H2, Mrg15, H3K36me3, H3K4me3, PolII pSer5, and PolII pSer2 across a region of chromosome 2L. (B) ChIP-qPCR validation of a subset of Cap-H2 ChIP-seq peaks using anti-Cap-H2 or IgG as negative control. IGV screenshot of corresponding ChIP-seq peaks are shown in Figure S5. Results are expressed as mean fold enrichment relative to control (RpL49) ± SEM of four biological replicates. *P < 0.05, Mann-Whitney rank sum test. (C–E and G–I) Salivary gland polytene chromosomes from wild-type larvae stained for Cap-H2 (red) and Pol IIo^Ser5^ or Pol IIo^Ser2^ (green) as indicated. (C, G) Enlarged split (D, H) and merged (E, I) images show overlap between Cap-H2 and Pol II staining. (F, J) Fluorescence intensity plots of images in E and I. RFU, relative fluorescence units.

Interbands are regarded as regions of open, decondensed chromatin whose level of chromatin compaction has been proposed to correlate with its level of transcriptional activity (Zhimulev *et al.* 2004). These regions are characterized by low nucleosomal density, DNAse I hypersensitivity, and the presence of markers associated with transcription, including phosphorylated RNA polymerase II (Pol II) (Zhimulev *et al.* 2014), and localization of Cap-H2 to these regions suggests some involvement in transcription. Furthermore, Mrg15 has been shown to act as a member of several chromatin-modifying complexes associated with transcription (Kusch *et al.* 2004; Pardo *et al.* 2002; Yochum and Ayer 2002), and it has been shown to associate with TSSs of active genes enriched in H3K4me3/2 and H3K9ac at the promoter region and H3K36me3 in gene bodies in *Drosophila* cells (Kharchenko *et al.* 2011). Therefore, we compared the genome-wide distribution of Cap-H2 in relation to marks of active transcription using previously published Cap-H2 ChIP-seq data and a number of modENCODE ChIP datasets (Celniker *et al.* 2009; Van Bortle *et al.* 2014). As observed by ChIP as well as in immunostaining images from polytene chromosomes (Figure 1), many binding sites of Cap-H2 overlap with regions bound by Mrg15 (Figure 2A). Interestingly, whereas Mrg15 is more broadly distributed, encompassing promoter regions and gene bodies, Cap-H2 appears as sharper peaks spanning promoter regions of genes (Figure 2A and Figure S3). As expected, Cap-H2 peak regions also correlate with regions enriched for H3K36me3, H3K4me3, and Pol IIo^Ser5^ and Pol IIo^Ser2^ (Figure 2A). To validate Cap-H2 Chip-seq peaks that overlap with Mrg15 and transcriptionally active marks, we selected a subset of peaks and confirmed enrichment of Cap-H2 at the selected target regions by ChIP-qPCR (Figure 2B).

To further confirm the association of Cap-H2 to transcriptionally active regions in other cell types, we performed immunostaining of polytene chromosomes with antibodies against phosphorylated forms of Pol II, Pol IIo^Ser5^, and Pol IIo^Ser2^. Cap-H2 co-localizes extensively with both Pol IIo^Ser5^ and Pol IIo^Ser2^ (76.3% and 73.3% overlap, respectively) (Figure 2, C–J and Figure S2). Although in lower magnification images some regions appear to have staining corresponding only to one of the proteins, higher magnification images reveal that the intensity of Cap-H2 at some sites correlates inversely with the intensity of Pol II so that the signal from one is obscured by the intensity of the other (Figure 2, D and H). This is particularly true for Pol IIo^Ser5^ (Figure 2D). Nevertheless, we rarely observed a Cap-H2 signal that did not overlap with Pol IIo^Ser5^ or Pol IIo^Ser2^, providing further evidence that Cap-H2 may be involved in regulation of gene transcription.

### Cap-H2 localization on polytene chromosomes is dependent on Mrg15

**Figure 3:**
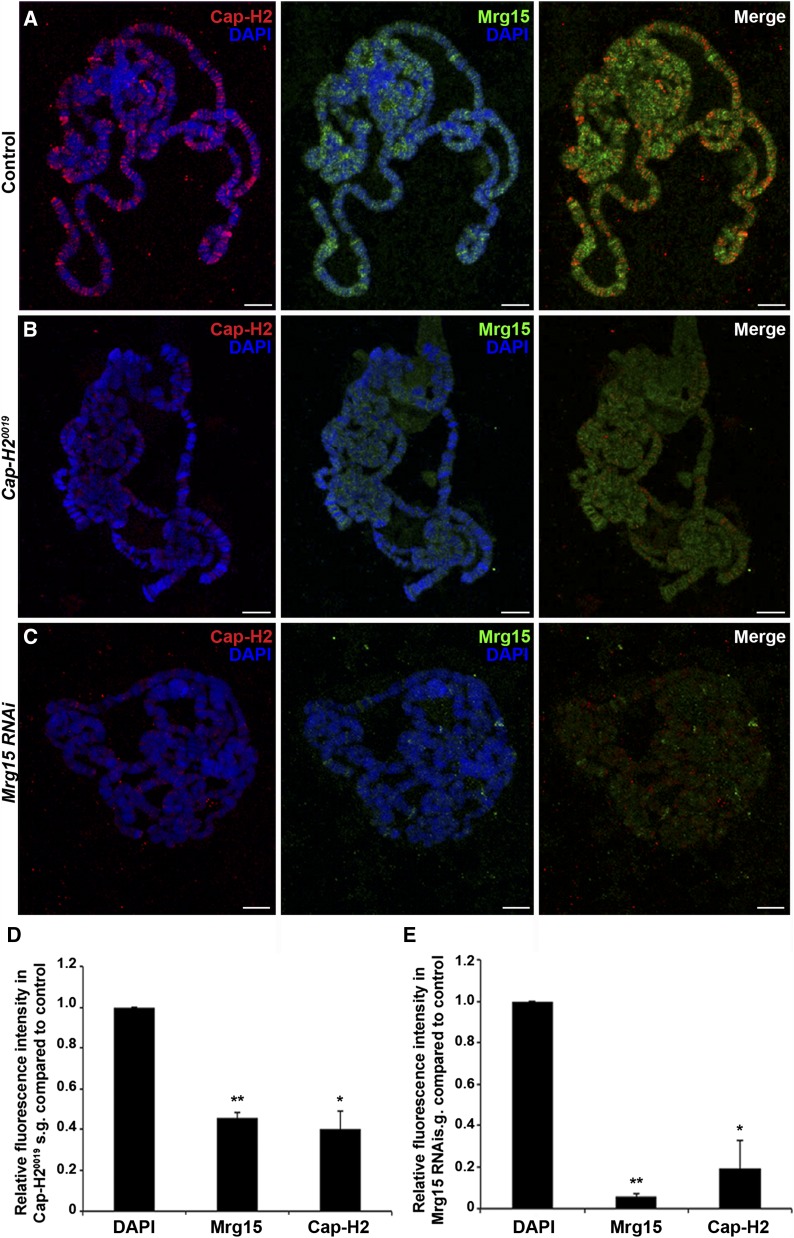
Mrg15 is required for Cap-H2 localization on polytene chromosomes. (A–C) Representative images of salivary gland polytene chromosomes from control (A), *Cap-H2^0019^* (B), and *Mrg15 RNAi* (C) larvae stained for Cap-H2 (red), Mrg15 (green), and DNA (blue). Scale bar, 10 μm. (D, E) Relative fluorescence intensity quantification showing significant decrease of Cap-H2 and Mrg15 in *Cap-H2^0019^* (D) and *Mrg15 RNAi* (E) polytene chromosomes. Mean fluorescence intensity was measured using four nuclei from each of three individual pairs of salivary glands and values were normalized to DAPI. *P < 0.05, **P < 0.01, two-tailed Student's *t*-test. Error bars, SEM.

To test if localization of Cap-H2 on chromatin is altered by loss of Mrg15, we performed immunofluorescence with Cap-H2 and Mrg15 antibodies on polytene chromosomes from salivary glands of larvae expressing *UAS > Mrg15 RNAi* under control of the salivary gland–specific *43B-Gal4* driver (Follette *et al.* 1998). Salivary glands from *43B-Gal4/+* control larvae and *43B-Gal4/+*; *UAS > Mrg15 RNAi/+* larvae were squashed side-by-side on the same microscope slide, as described previously (Pal-Bhadra *et al.* 2004), and images were acquired under identical conditions to compare relative levels of each protein on the chromosomes from each genotype. Immunostaining of *Cap-H2^0019^* polytene chromosomes showed a significant decrease in Cap-H2 fluorescence compared with OR control (Figure 3, A, B, and D), confirming our ability to detect quantifiable changes in the fluorescence intensity of Cap-H2 as well as the specificity of the antibody. Similarly, chromosomes from larvae expressing *Mrg15 RNAi* showed an almost complete loss of Mrg15 staining, confirming that Mrg15 was depleted in salivary glands obtained from these larvae (Figure 3, C and E). Cap-H2 fluorescence levels were also reduced significantly, to approximately 20% of control (Figure 3, C and E), with weak signal remaining at a small number of sites along the chromosome arms. This finding is consistent with previous chromatin fractionation data (Smith *et al.* 2013), providing further evidence that binding of Cap-H2 on chromatin is partially dependent on Mrg15. Interestingly, Mrg15 fluorescence levels were reduced to approximately half of control in polytene chromosomes from *Cap-H2^0019^* larvae, suggesting that there may be some level of interdependence of binding between Cap-H2 and Mrg15. We observed a similar loss of Cap-D3 binding on polytenes from *Mrg15 RNAi* larvae (Figure S4), suggesting that Mrg15 is required for proper localization of the entire condensin II complex on chromatin.

### Cap-H2 interaction with Mrg15 is dependent on its Mrg-binding motif

**Figure 4:**
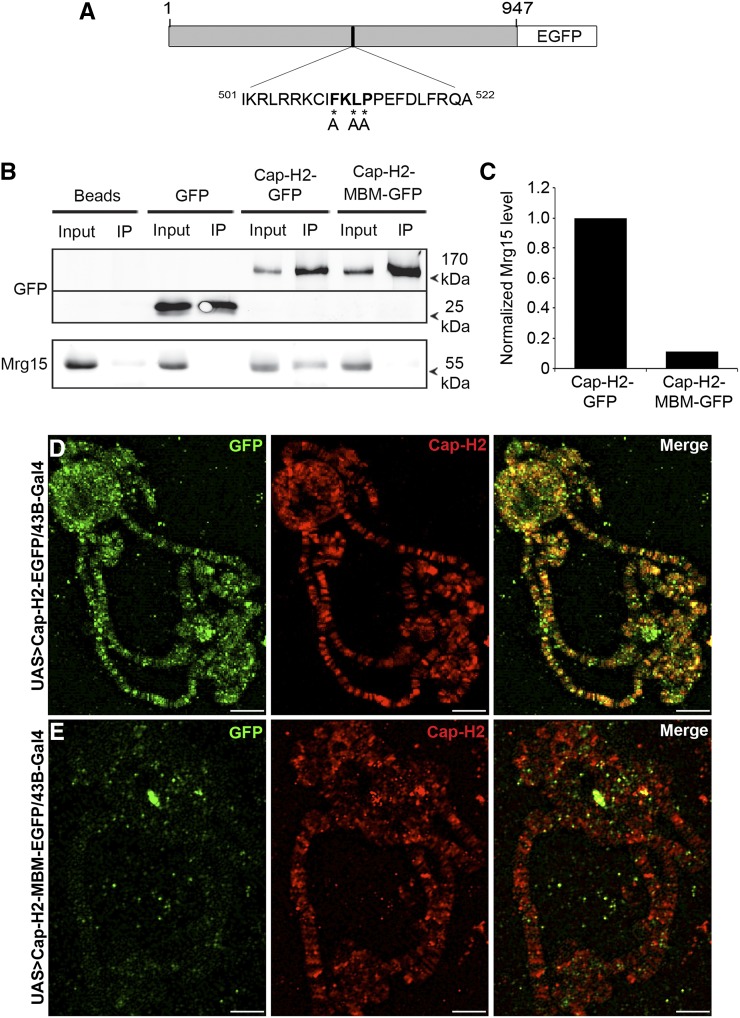
Mrg-binding motif is required for Cap-H2 interaction with Mrg15. (A) Schematic representation of the Cap-H2-MBM-EGFP protein. Numbers represent amino acid positions relative to the full-length 947-amino-acid Cap-H2 protein sequence. FKLP sequence is shown in bold and asterisks indicate residues mutated to alanine (residues 510, 512-513). (B) Mutation of the Cap-H2 Mrg-binding motif results in loss of Cap-H2 association with Mrg15. GFP-binding protein immunoprecipitates from lysates of S2 cells transfected with pMT-Cap-H2-EGFP or pMT-Cap-H2-MBM-EGFP immunoblotted for anti-GFP (top) and anti-Mrg15 (bottom) antibodies. (C) Normalized densitometric ratio of Mrg15 bands relative to corresponding Cap-H2-GFP or Cap-H2-MBM-GFP bands for the representative Western blot shown in (B). Salivary gland polytene chromosomes from *UAS > Cap-H2-EGFP/43B-Gal4* (D) and *UAS > Cap-H2-MBM-EGFP/43B-Gal4* (E) larvae immunostained for Cap-H2 (red) and GFP (green). Scale bar, 10 μm.

Recent structure–function analysis of the MRG domain of MRG15 had identified an Mrg15 protein-binding consensus sequence (FxLP) (Xie *et al.* 2012). We previously found that all four reported *Drosophila* Cap-H2 protein isoforms contain an FKLP sequence (Smith *et al.* 2013), which is unique to Cap-H2 and does not occur in the other condensin II subunits. To assess whether this sequence is important for the interaction between Cap-H2 and Mrg15, we used site-directed mutagenesis to generate a Cap-H2 Mrg-binding motif mutant (Cap-H2-MBM-EGFP). Three of the four residues of the consensus sequence were mutated to alanines, changing the FKLP sequence to AKAA (F510A, L512A, P513A) (Figure 4A). The interaction between mutant Cap-H2 and Mrg15 was tested by immunoprecipitation in S2 cells transiently transfected with pMT-Cap-H2-MBM-EGFP. We detected the presence of both Cap-H2-EGFP and endogenous Mrg15 in GFP-binding protein immunoprecipitates from cells expressing wild-type Cap-H2-EGFP, but not in immunoprecipitates from cells expressing EGFP alone (Figure 4B, top panel). Cap-H2-MBM-EGFP also immunoprecipitated with GFP-binding protein (Figure 4B, top panel); however, we could not detect the presence of endogenous MRG15 in immunoprecipitates from cells expressing the mutant Cap-H2 (Figure 4B, bottom panel). Quantification of bands from the Western blot shown in Figure 4B showed that Mrg15 levels were reduced by 89% in cells expressing Cap-H2-MBM-EGFP relative to cells expressing Cap-H2- EGFP (Figure 4C). This result indicates that the FKLP sequence in Cap-H2 is a genuine Mrg15-binding motif and that the interaction between Cap-H2 and MRG15 is mediated by the Mrg-binding motif.

Previously, knockdown of Mrg15 was shown to result in partial loss of Cap-H2 binding on chromatin in S2 cells, suggesting that Mrg15 facilitates Cap-H2 binding or recruitment to chromosomes (Smith *et al.* 2013). Because the Mrg-binding motif in Cap-H2 is required for its interaction with Mrg15, it would be expected that mutation of this sequence would alter binding of Cap-H2 to chromatin. We therefore generated transgenic flies expressing eGFP-tagged Cap-H2 or Cap-H2-MBM under control of the UASt promoter (Rorth 1998) to visualize these proteins on polytene chromosomes. We first overexpressed Cap-H2-EGFP in larval salivary glands *43B-Gal4* to drive expression of the transgenes in larval salivary glands. Immunostaining of polytene chromosomes from *43B-Gal4/UAS > Cap-H2-eGFP* salivary glands using anti-Cap-H2 and anti-GFP antibodies revealed that Cap-H2-EGFP co-localizes extensively with endogenous Cap-H2, indicating functionality of the fusion protein (Figure 4D). Although the Cap-H2 antibody can detect both endogenous and GFP-tagged protein, anti-GFP staining is specific for the tagged protein; therefore, it is important to note that we did not observe any instances in which we could detect an anti-Cap-H2 band that did not have a corresponding GFP signal. When Cap-H2-MBM-EGFP was expressed, we were no longer able to detect anti-GFP fluorescence on chromosomes at sites where endogenous Cap-H2 localizes (Figure 4E), indicating that interaction with Mrg15 via the Mrg-binding motif is necessary for the proper localization of Cap-H2 on chromatin. We note that failure to localize to chromatin is likely not due to instability of this Cap-H2-MBM-EGFP protein because expression in cultured cells appears to be robust and comparable with Cap-H2-EGFP, relative to endogenous Mrg15 protein levels (Figure 4B).

### Mrg-binding motif is required for Cap-H2-mediated homolog unpairing and axial compaction in cultured cells

**Figure 5:**
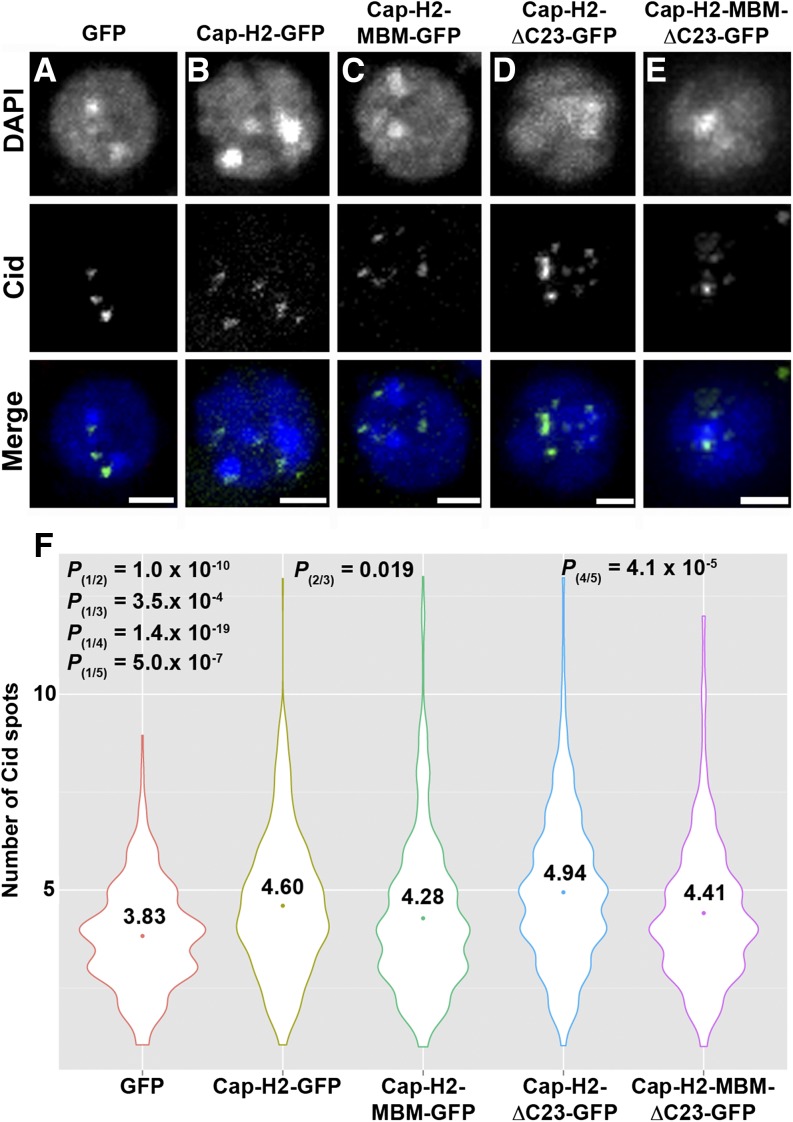
Mrg-binding motif is required for Cap-H2-mediated dispersal of pericentric heterochromatin. (A–F) Mutation of Mrg-binding motif (MBM) suppresses Cap-H2-mediated Cid dispersal. Kc cells transiently expressing EGFP (A), Cap-H2-EGFP (B), Cap-H2-MBM-EGFP (C), Cap-H2-ΔC23-EGFP (D), or Cap-H2-ΔC23-MBM-EGFP (E) stained for Cid (green) and DNA (blue). Scale bar, 2.5 μm. (F) Violin plots showing the number of Cid spots per nucleus in Kc cells expressing EGFP (1) (n = 320), Cap-H2-EGFP (2) (n = 370), Cap-H2-MBM-EGFP (3) (n = 370), Cap-H2-ΔC23-EGFP (4) (n = 417), or Cap-H2-ΔC23-MBM-EGFP (5) (n = 417). Colored circle, mean value; *P* = *P* value, two-tailed Student's *t*-test; numbers in parentheses indicate pairwise comparisons.

Condensin II promotes unpairing of homologous chromosomes, a process that has been proposed to result from condensin II–mediated axial compaction (Bauer *et al.* 2012; Joyce *et al.* 2012). Overexpression of Cap-H2 is sufficient to drive compaction and unpairing in interphase chromosomes, and does so in an Mrg15-dependent manner (Bauer *et al.* 2012; Smith *et al.* 2013). To determine whether the Cap-H2 Mrg-binding motif is required for its compaction and antipairing functions, we first set out to determine whether the Mrg-binding motif is required for Cap-H2–mediated centromeric dispersal. To test this, we immunostained Kc cells using an antibody against the centromere identifier protein (Cid) and counted the number of Cid spots in cells transfected with wild-type and MBM mutant Cap-H2-EGFP constructs. Kc cells were chosen for these analyses, because this cell line was used previously to assess chromosome pairing to avoid any possible effect of the segmental aneuploidy of S2 cells on pairing measurements (Buster *et al.* 2013). Kc cells are tetraploid and exhibit a high degree of pairing (Williams *et al.* 2007). The number of Cid spots observed per cell is indicative of the level of pairing of homologous centromeric regions, with a lower number of Cid spots signifying a more paired state and a higher number signifying unpairing. Control cells transfected with pMT-EGFP had 3.83 ± 0.07 (mean ± SEM) Cid spots per cell (Figure 5, A and F, n = 320). Expression of Cap-H2-EGFP induced Cid dispersal, with the number of Cid spots increasing over control to 4.6 ± 0.09 per cell (Figure 5, B and F, n = 370). Expression of Cap-H2-MBM-EGFP led to a decrease in the number of Cid spots (4.28 ± 0.10, n = 370) compared with cells expressing Cap-H2-EGFP (Figure 5, C and F). We also transfected cells with a stable form of Cap-H2 (Cap-H2-ΔC23-EGFP) containing a deletion of the C-terminal 23 amino acids that prevents its degradation by the E3 ubiquitin ligase Slimb, thus leading to accumulation of high levels of Cap-H2 and an increase in severity of Cap-H2–mediated compaction and unpairing phenotypes (Buster *et al.* 2013). Expression of Cap-H2-ΔC23-EGFP induced even greater Cid dispersal than wild-type Cap-H2-EGFP (Figure 5, D and F), and that was partially suppressed by expression of Cap-H2-MBM-ΔC23-EGFP containing a mutation in the Mrg-binding motif (4.94 ± 0.09 and 4.41 ± 0.09, respectively; n= 417) (Figure 5, E and F). The average number of Cid spots per nucleus differs from what has been previously reported for pMT-EGFP and Cap-H2-ΔC23-EGFP (7.2 and 14.4, respectively) in S2 cells, and may be attributed to choice of cell line and the variability in phenotypic strength among various cell types, which has been noted previously (Buster *et al.* 2013). However, our results from pMT-EGFP transfected cells (3.83 spots per cell) are consistent with our observations from control RNAi-treated Kc cells (3.63) (Nguyen *et al.* 2015), as well as previous findings that Cid staining is visible in approximately four to six spots per nucleus in Kc cells (Ahmad and Henikoff 2001; Henikoff *et al.* 2000).

**Figure 6:**
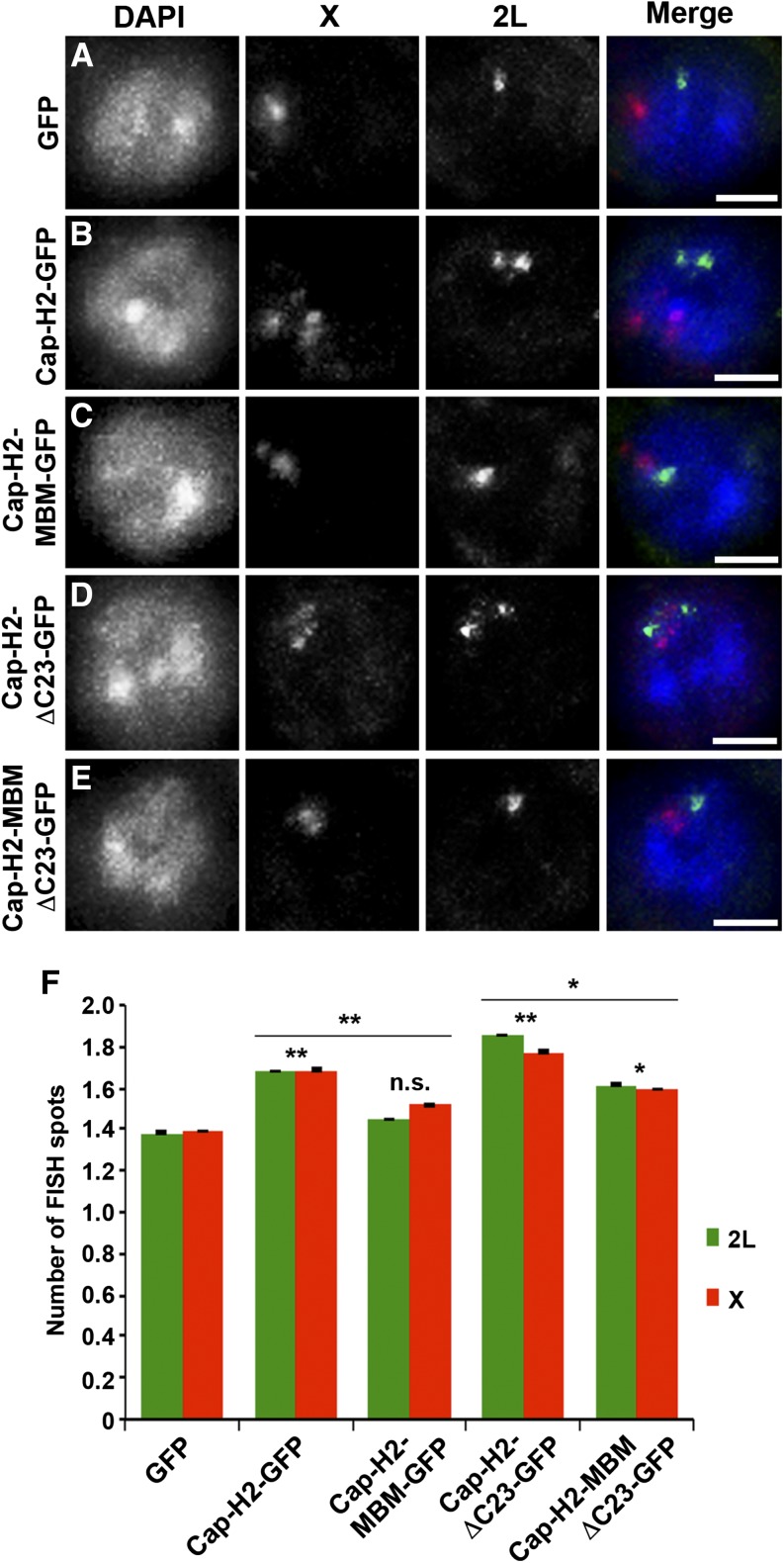
Mrg-binding motif is required for Cap-H2-mediated unpairing of homologous chromosomes. Kc cells transiently expressing EGFP (A), Cap-H2-EGFP (B), Cap-H2-MBM-EGFP (C), Cap-H2-ΔC23-EGFP (D), or Cap-H2-ΔC23-MBM-EGFP (E) labeled with DNA FISH probes to the 2L (green) and X chromosomes (red). Scale bar, 2.5 µm. (F) Number of FISH spots per nucleus in Kc cells transfected as in (A–E). At least 145 cells were counted for each category shown. *P < 0.05, **P < 0.01, two-tailed Student's *t*-test. *P* values correspond to statistical significance relative to control, except where indicated by horizontal lines. Asterisks located above horizontal black line indicate significance between cells expressing Cap-H2-EGFP or Cap-H2-ΔC23-EGFP and the corresponding MBM mutant. Error bars, SEM; n.s. = nonsignificant.

We further analyzed the role of the Cap-H2 Mrg-binding domain in homologous chromosome unpairing using FISH in Kc cells. We designed probes to label euchromatic loci on chromosomes X and 2L, and we assessed the level of pairing by counting the number of FISH spots per nucleus. Cap-H2 overexpression drives chromosome unpairing in Kc cells, as indicated by an increase in the number of observable FISH spots, a phenotype that is suppressed by depletion of Mrg15 (Smith *et al.* 2013). Similarly, we observed a significant increase in the number of FISH spots per nucleus of Cap-H2-EGFP expressing cells (X = 1.69 ± 0.71 and 2L = 1.68 ± 0.84; n = 145) relative to control (X = 1.39 ± 0.57 and 2L = 1.38 ± 0.59; n = 146) (Figure 6, A, B, and F). Expression of Cap-H2-MBM-EGFP results in reduction of FISH spots (X = 1.52 ± 0.54 and 2L = 1.45 ± 0.57; n = 147) relative to Cap-H2-EGFP to a level similar to control (Figure 6, C and F). Consistent with previous observations, expression of Cap-H2-ΔC23-EGFP led to a further increase in unpairing (Figure 6, D and F) (Buster *et al.* 2013). The number of FISH spots also decreased significantly in cells expressing Cap-H2-MBM-ΔC23-EGFP (X = 1.60 ± 0.61 and 2L = 1.61 ± 0.63; n = 147) relative to Cap-H2-ΔC23-EGFP (X = 1.77 ± 0.83 and 2L = 1.85 ± 0.80; n = 147), although the number of FISH spots remained elevated compared with control (Figure 6, D–F). Taken together, both the FISH and CID dispersal data consistently show that mutation in the Mrg-binding motif of Cap-H2 results in a significantly less robust anti-pairing activity, indicating that the Cap-H2 Mrg-binding motif is critical for its function. This suggests that the Cap-H2-Mrg15 protein–protein interaction, via the Mrg-binding motif, is important for condensin II–mediated chromosome anti-pairing activity.

**Figure 7:**
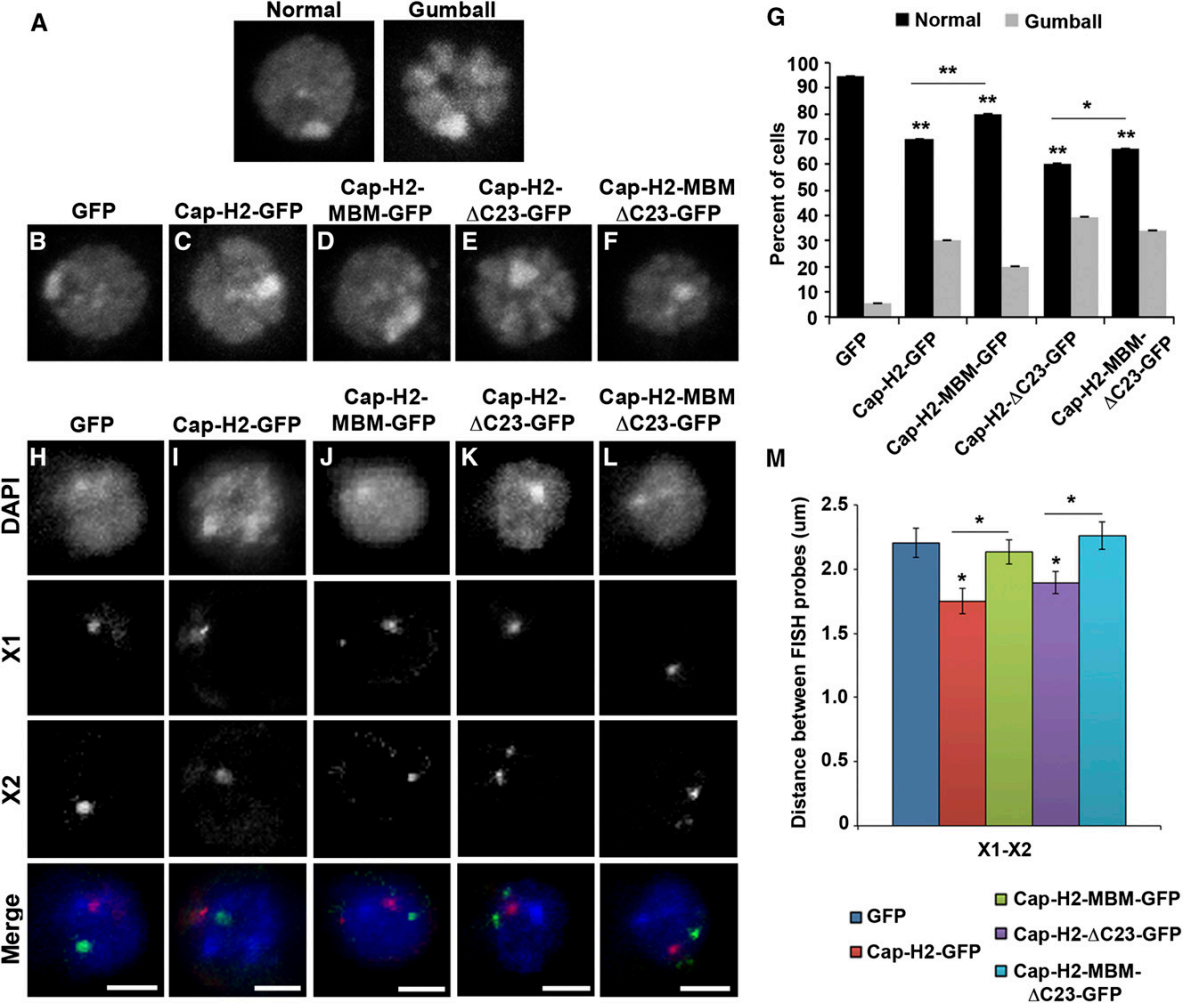
Mrg-binding motif is required for Cap-H2-mediated axial compaction. (A) DNA-stained Kc cells displaying representative chromatin gumball phenotypes after overexpression of Cap-H2-ΔC23-EGFP. DNA-stained Kc cells transiently expressing EGFP (B), Cap-H2-EGFP (C), Cap-H2-MBM-EGFP (D), Cap-H2-ΔC23-EGFP (E), or Cap-H2-ΔC23-MBM-EGFP (F). (G) Percentage of Kc cells transfected as in (B–F) exhibiting the chromatin gumball phenotype. *P < 0.05, **P < 0.001, two-tailed Fisher’s exact test. Error bars represent 95% C.I.; a minimum of 500 cells were counted for each category. Kc cells transiently expressing EGFP (H), Cap-H2-EGFP (I), Cap-H2-MBM-EGFP (J), Cap-H2-ΔC23-EGFP (K), or Cap-H2-ΔC23-MBM-EGFP (L) labeled with FISH probes for two X chromosome loci, X chromosome probe 1 (X1, red) and X chromosome probe 2 (X2, green). Scale bar, 2.5 µm. (M) Pairwise distances were measured for X1–X2 FISH probes (n = 60). *P < 0.05, two-tailed Student's *t*-test. *P* values correspond to statistical significance relative to control, except where indicated by horizontal lines. Asterisks located above horizontal black line indicate significance between cells expressing Cap-H2-EGFP or Cap-H2-ΔC23-EGFP and the corresponding MBM mutant. Error bars, SEM.

Overexpression of Cap-H2 both *in vivo* and in *Drosophila* cultured cells results in a reorganization of the interphase nucleus, resulting in hypercompaction of chromosomes into multiple globular structures that we refer to as the “chromatin gumball” phenotype (Figure 7A) (Buster *et al.* 2013). These chromatin gumballs are reminiscent of chromosome territories, which have been proposed to occur as a consequence of condensin II–mediated chromosome compaction (Bauer *et al.* 2012). We tested whether the Mrg-binding domain of Cap-H2 is necessary for this remodeling of the interphase nucleus by observing chromatin organization of DAPI-stained nuclei in cells expressing GFP-tagged Cap-H2 constructs and their corresponding Mrg-binding mutants for the presence of the gumball phenotype. Expression of Cap-H2-EGFP and Cap-H2-ΔC23-EGFP resulted in increasingly strong chromatin-gumball phenotypes in Kc cells, with 30% of Cap-H2-EGFP expressing cells and 39% of Cap-H2-ΔC23-EGFP expressing cells exhibiting this phenotype (Figure 7, C, E, and G). Expression of Cap-H2-MBM-EGFP and Cap-H2-MBM-ΔC23-EGFP resulted in partial suppression of chromosome reorganization, with a reduction to 20% and 34% of cells exhibiting gumball formation, respectively.

To further assess the effects of the Mrg-binding motif of Cap-H2 on axial compaction, we performed 3D DNA FISH in Kc cells using two probes each for euchromatic regions located approximately 2 Mb apart on chromosomes X and 2L (Figure 7, H–M and Figure S5). Distances between probes located on each chromosome were measured to assess the degree of chromosome compaction. Expression of Cap-H2-EGFP resulted in a significant decrease in pairwise distances between sets of FISH probes on each chromosome (X1–X2 = 1.75 ± 0.10 μm and 2L1–2L2 = 0.96 ± 0.04 μm; n = 60) when compared with control cells expressing EGFP (X1–X2 = 2.20 ± 0.15 and 2L1–2L2 = 1.21 ± 0.05 μm;n = 60) (Figure 7, H, I, and M). Similar to the effects of depletion of Mrg15 (Smith *et al.* 2013), expression of Cap-H2-MBM-EGFP suppressed chromosome compaction, resulting in probe distances similar to control (X1–X2 = 2.13 ± 0.10 μm and 2L1–2L2 = 1.11 ± 0.05 μm; n = 60) (Figure 7, J and M). Similarly, Cap-H2-ΔC23-EGFP expression promoted an increase in axial compaction (X1–X2 = 1.89 ± 0.08 μm and 2L1–2L2 = 1.01 ± 0.04 μm; n = 60) comparable to that seen in cells expressing Cap-H2-EGFP when compared with control (Figure 7, K and M). Likewise, we observed suppression of axial compaction in cells expressing Cap-H2-MBM-ΔC23-EGFP relative to Cap-H2-ΔC23-EGFP (X1–X2 = 2.26 ± 0.11 μm and 2L1–2L2 = 1.16 ± 0.04 μm; n = 60) (Figure 7, L and M). Taken together, these data indicate that the Mrg-binding motif of Cap-H2 is required for Cap-H2 axial compaction activity. This suggests that the Cap-H2 interaction with Mrg15 is critical for facilitating condensin II–mediated interphase chromosome compaction.

### Mrg-binding motif is required for Cap-H2-mediated unpairing of salivary gland chromosomes

**Figure 8:**
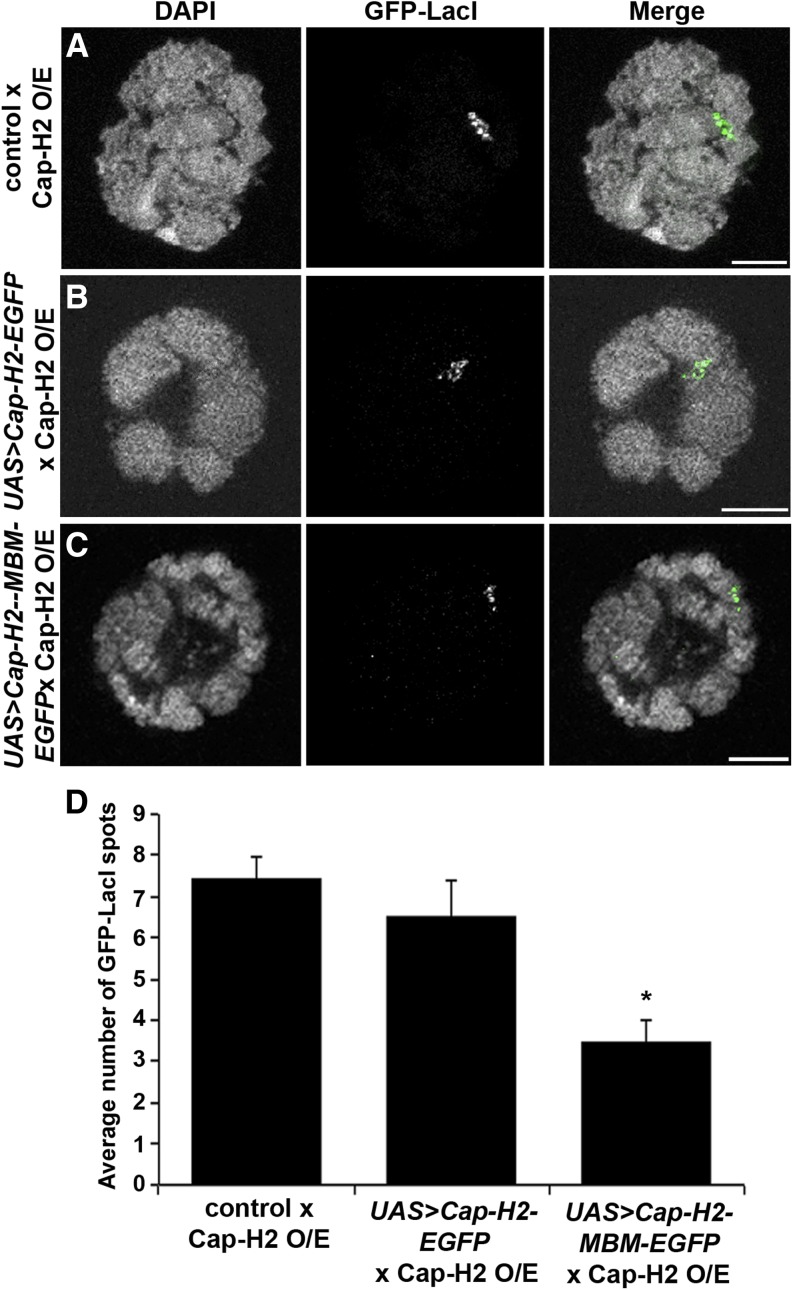
Overexpression of Mrg-binding motif mutant suppresses Cap-H2-mediated unpairing of salivary gland chromosomes. Salivary gland nuclei of heat-shocked larvae from Cap-H2 pairing reporter (O/E) flies crossed to *yw* (A), UAS > Cap-H2-EGFP (B), and UAS > Cap-H2-MBM-EGFP (C) showing DNA (DAPI, grayscale) and GFP-LacI (green). Scale bar, 10 µm. (D) Average number of GFP-LacI spots per nucleus. *P < 0.001, two-tailed Student's *t*-test.

To determine whether the Mrg-binding motif is important for condensin II function *in vivo*, we tested its requirement for dispersal of salivary gland polytene chromosomes. We have previou**s**ly shown that Cap-H2 overexpression in the larval salivary gland is sufficient to drive polytene chromosome unpairing, and that Mrg15 is required for this to occur *in vivo* (Hartl *et al.* 2008; Smith *et al.* 2013). To test whether this phenotype is mediated by the Mrg-binding motif, we used w[*]; P(w[+mC] = lacO.256x)60F, *hs83 > GFP-LacI*; *Hsp70 > Gal4*, *Cap‐H2^EY09979^* transgenic pairing reporter flies, which contain an insertion of a LacO array inserted in the second chromosome at cytological region 60F, a heat-shock inducible GFP-LacI, as well as GAL4 to drive overexpression of wild-type Cap-H2 (UAS > Cap-H2^EY09979^), as described previously (Hartl *et al.* 2008). Although salivary gland polytene chromosomes are normally tightly paired, overexpression of Cap-H2 on heat shock induction drives unpairing of chromosomes, which can be visualized as numerous GFP spots due to binding of LacI-GFP proteins to the unpaired LacO array sequences. This process requires endogenous SMC2/4 and Cap-D3, indicating that Cap-H2 likely drives chromosome unpairing within the context of the condensin II complex (Buster *et al.* 2013; Hartl *et al.* 2008; Smith *et al.* 2013). To determine if the Mrg-binding motif is important for Cap-H2-driven polytene chromosome unpairing, we crossed the pairing reporter flies to UAS > Cap-H2-EGFP or UAS > Cap-H2-MBM-EGFP transgenic flies to generate offspring with the genotypes P(w[+mC] = lacO.256x)60F, *hs83 > GFP-LacI/ UAS > Cap-H2-EGFP*; *Hsp70 > Gal4*, *Cap‐H2^EY09979^*/+ or P(w[+mC] = lacO.256x)60F, *hs83 > GFP-LacI/ UAS > Cap-H2-MBM-EGFP*; *Hsp70 > Gal4*, *Cap‐H2^EY09979^*/+. These flies were used to overexpress Cap-H2 using the Cap-H2 *^EY09979^* allele in the background of either the UAS > Cap-H2-EGFP or the UAS > Cap-H2-MBM-EGFP constructs (Figure 8). If the Mrg-binding motif is required to drive chromosome unpairing, then we would expect that Cap-H2-MBM-EGFP might compete with wild-type Cap-H2 for association with other components of the condensin II complex, therefore reducing the level of chromatin-bound condensin II and leading to suppression of chromosome unpairing. When the pairing reporter flies were crossed with UAS > Cap-H2-EGFP, polytene chromosomes from salivary glands of these offspring were unpaired after heat shock, with nuclei exhibiting a similar number of LacI-GFP spots compared with offspring from the control cross (6.5 ± 0.90 *vs.* 7.4 ± 0.50, respectively) (Figure 8, A, B, and D). However, salivary glands from larvae overexpressing both Cap-H2 and Cap-H2-MBM-EGFP exhibited a higher degree of pairing, as indicated by the significant decrease in LacI-GFP spots (3.5 ± 0.48) relative to glands from control and Cap-H2-EGFP expressing larvae (Figure 8, C and D). These results indicate that the Cap-H2 Mrg-binding motif is required *in vivo* for condensin II–mediated polytene chromosome dispersal, and Cap-H2 molecules with mutations in this motif can suppress gain-of-function phenotypes produced by overexpression of wild-type Cap-H2. Taken together, our results suggest that the Mrg-binding motif of Cap-H2 mediates interaction of condensin II and Mrg15 at transcriptionally active regions of chromatin to facilitate interphase chromosome compaction and unpairing.

## Discussion

In this study, we have shown that the Cap-H2 subunit of condensin II binds to interband regions of polytene chromosomes in an Mrg15-dependent manner. Cap-H2 localizes to gene promoters in regions of chromatin that are enriched for factors associated with active transcription, including Mrg15, H3K36me3, H3K4me3, and Pol II. We have identified an FKLP Mrg-binding motif sequence within Cap-H2 that is required for interaction of Cap-H2 with Mrg15 and localization of Cap-H2 on polytene chromosomes. Furthermore, the Mrg-binding motif is required for condensin II–mediated chromatin compaction in cultured cells and homolog unpairing in cells and *in vivo*. These findings support a model in which Mrg15 acts as a loading factor for Cap-H2 to recruit condensin II to transcriptionally active chromatin to facilitate its compaction and unpairing activities.

We have previously shown that Mrg15 is required for condensin II–mediated maintenance of interphase compaction and homolog pairing and that Cap-H2 and Mrg15 physically interact through the MRG domain of Mrg15 (Smith *et al.* 2013). Based on these observations, we proposed that Mrg recruits Cap-H2 to chromatin to facilitate condensin II activity on chromatin, although it was unclear whether this interaction occurred directly or indirectly through another component of an Mrg15-containing complex, such as Tip60. Here, we have identified an Mrg-binding motif within Cap-H2 that matches a predicted Mrg15 protein-binding consensus sequence (FxLP) derived from structure–function studies of the MRG domain as well as sequence analysis of Mrg15 interactors (Xie *et al.* 2012). This FxLP sequence was shown to be conserved among several known Mrg15 interactors, including Pf1, PAM14, and PALB2, members of the mSin3-HDAC, MAF1, and BRCA complexes, respectively, each of which interacts directly with Mrg15 (Sy *et al.* 2009; Xie *et al.* 2012; Yochum and Ayer 2002; Zhang *et al.* 2006b). Furthermore, the FxLP sequence was determined to be necessary, although not sufficient, for high-affinity interaction between Mrg15 and Pf1 (Xie *et al.* 2012), suggesting that this sequence facilitates direct interaction between Mrg15 and its binding partners. Considering these observations, our finding that Cap-H2 similarly requires the FKLP Mrg-binding motif sequence for interaction with Mrg15 and proper localization on polytene chromosomes provides support for a direct role for Mrg15 in recruitment or tethering of condensin II to chromatin through direct protein–protein interactions between the MRG domain of Mrg15 and the Mrg-binding motif of Cap-H2. The FxLP motif sequence identified within Cap-H2 is not well-conserved among metazoans; therefore, it will be interesting to determine whether the condensin-recruiting function of Mrg15 is conserved. It is possible that the functional interaction between Cap-H2 and Mrg15 is conserved in other organisms, but that other regions of the Cap-H2 protein mediate the interaction or that Cap-H2 may be part of a complex containing Mrg15 and may interact with it only in an indirect manner.

The finding that localization of Cap-H2 on salivary gland polytene chromosomes is dependent on Mrg15 and requires an intact Mrg-binding motif lends further support to the idea that Mrg15 acts to recruit or tether condensin II to chromatin. Interestingly, mutation of Cap-H2 results in decrease of Mrg15 binding, indicating possible cooperation of binding and raising the possibility that Cap-H2 interaction with Mrg15 may be necessary to stabilize Mrg15 on chromatin. The partial dependence of Cap-H2 on Mrg15 for proper localization as well as our previous observations that binding of Cap-H2 on chromatin in cultured cells is partially dependent on Mrg15 indicates that there may be other as yet unidentified proteins that may also be important for localization of condensin II to chromatin. We cannot rule out that the partial loss of chromatin-bound Cap-H2 is due to incomplete RNAi depletion of Mrg15. However, our functional assays in cells overexpressing Cap-H2 protein with a mutated Mrg-binding motif also show only partial dependence on interaction with Mrg15, because this mutant Cap-H2 was unable to completely suppress condensin II–mediated unpairing and compaction to control levels. An alternative explanation for these observations in cells that are expressing mutant Cap-H2 may be that while mutation of the Mrg-binding motif abolishes the interaction of Cap-H2 with Mrg15, this mutant Cap-H2 might still be incorporated into partially functional condensin II complexes on chromatin, either by interaction with other condensin II components or by dimerization with endogenous wild-type Cap-H2 molecules. However, the loss of Cap-D3 localization on polytene chromosomes on depletion of Mrg15 argues against this possibility (Figure S4). Furthermore, suppression of polytene unpairing by overexpression of MBM mutant Cap-H2 in larvae that are also overexpressing wild-type Cap-H2 raises the possibility that the MBM mutant competes with wild-type Cap-H2 for incorporation into chromatin-bound condensin II complexes. Replacement of wild-type Cap-H2 by MBM mutant Cap-H2 might result in loss of interaction with Mrg15 and subsequent reduction of complex localization on chromatin, supporting the possibility of Mrg15-independent condensin II function. Further work will be necessary to differentiate between these possibilities. Nevertheless, our results clearly show that interaction of Cap-H2 with Mrg15 via the Mrg-binding motif is required for condensin II chromosome compaction and unpairing activity.

The presence of Cap-H2 and Cap-D3 at interband regions of polytene chromosomes enriched for Pol II and its localization in cultured cells near promoter regions of genes enriched in marks of active chromatin is consistent with previous studies of condensin complex localization in *C. elegans* and in mouse embryonic stem cells (ESCs). The first reported genome-wide analysis of metazoan condensin II was performed in *C. elegans*, where it was shown that condensin I, condensin I^DC^, the condensin-like *C. elegans* dosage compensation complex, and condensin II are all enriched at active enhancers and promoters (Kranz *et al.* 2013). Mammalian condensin II also binds active enhancers and promoters in mouse ESCs, where its enrichment correlates with that of Pol II at genes (Dowen *et al.* 2013). Condensin II binds to the same regions as cohesin and, like cohesin enrichment of condensin II in ESCs, was reduced on shRNA knockdown of the cohesin loading factor NIPBL (Dowen *et al.* 2013). It remains unclear, however, whether the role of NIPBL in loading of condensin II on chromatin is direct or if altered loading of cohesin affects condensin II chromatin levels. In *C. elegans*, a DNA sequence was identified as a putative condensin complex recruitment motif, but no factors have been identified that are important for targeting of condensins to chromatin in worms. Similarly, it is not known whether other condensin loading factors, such as NIPBL, exist in *Drosophila*. However, it is interesting to note that Mrg-1, the worm Mrg15 homolog, plays a role in homologous chromosome pairing during meiosis (Dombecki *et al.* 2011) and that, like the condensin-like DCC, it silences X-linked genes (Takasaki *et al.* 2007), suggesting that Mrg15 may play a conserved role in targeting of condensin complexes to chromatin and their function in other organisms.

Binding of Cap-H2 at active enhancers and promoters suggests that condensin II may function to regulate gene transcription. Previous observations for *Drosophila* Cap-D3 as well as mouse and worm condensin II support a conserved role for condensins in transcriptional regulation (Dowen *et al.* 2013; Kranz *et al.* 2013; Longworth *et al.* 2012). Condensin complexes have been proposed to function as transcriptional repressors based on their role in rRNA gene transcription in yeast and mammalian cells and in gene repression in *C. elegans*, particularly in dosage compensation (Huang *et al.* 2013; Kranz *et al.* 2013; Machin *et al.* 2004). In *Drosophila*, Cap-D3 and RBF1 co-regulate genes in a stage-specific manner, either activating or repressing certain sets of genes at different times during development (Longworth *et al.* 2012). Mrg15 has been shown to act as both a transcriptional repressor and activator, depending on the other chromatin remodeling complex factors with which it interacts (Cai *et al.* 2003; Pardo *et al.* 2002; Yochum and Ayer 2002). Determining whether Cap-H2 and Mrg15 function within the same multi-protein complex to coordinately regulate transcription of target genes and whether they do so in a cell type–specific or developmental stage–specific manner will provide valuable insight into the function of condensin complexes in maintenance of interphase genome organization and their contribution to proper control of gene expression.
